# FRA-1 protein overexpression is a feature of hyperplastic and neoplastic breast disorders

**DOI:** 10.1186/1471-2407-7-17

**Published:** 2007-01-25

**Authors:** Gennaro Chiappetta, Angelo Ferraro, Gerardo Botti, Mario Monaco, Rosa Pasquinelli, Emilia Vuttariello, Liliane Arnaldi, Maurizio Di Bonito, Giuseppe D'Aiuto, Giovanna Maria Pierantoni, Alfredo Fusco

**Affiliations:** 1 Istituto Nazionale dei Tumori di Napoli Fondazione Senatore Pascale, Naples, Italy; 2 NOGEC (Naples Oncogenomic Center)-CEINGE Biotecnologie Avanzate-Napoli & SEMM-European School of Molecular Medicine-Naples Site, Naples, Italy; 3 Dipartimento di Biologia e Patologia Cellulare e Molecolare, Università di Napoli "Federico II", Naples, Italy

## Abstract

**Background:**

Fos-related antigen 1 (FRA-1) is an immediate early gene encoding a member of AP-1 family of transcription factors involved in cell proliferation, differentiation, apoptosis, and other biological processes. *fra-1 *gene overexpression has an important role in the process of cellular transformation, and our previous studies suggest FRA-1 protein detection as a useful tool for the diagnosis of thyroid neoplasias. Here we investigate the expression of the FRA-1 protein in benign and malignant breast tissues by immunohistochemistry, Western blot, RT-PCR and qPCR analysis, to evaluate its possible help in the diagnosis and prognosis of breast neoplastic diseases.

**Methods:**

We investigate the expression of the FRA-1 protein in 70 breast carcinomas and 30 benign breast diseases by immunohistochemistry, Western blot, RT-PCR and qPCR analysis.

**Results:**

FRA-1 protein was present in all of the carcinoma samples with an intense staining in the nucleus. Positive staining was also found in most of fibroadenomas, but in this case the staining was present both in the nucleus and cytoplasm, and the number of positive cells was lower than in carcinomas. Similar results were obtained from the analysis of breast hyperplasias, with no differences in FRA-1 expression level between typical and atypical breast lesions; however the FRA-1 protein localization is mainly nuclear in the atypical hyperplasias. *In situ *breast carcinomas showed a pattern of FRA-1 protein expression very similar to that observed in atypical hyperplasias. Conversely, no FRA-1 protein was detectable in 6 normal breast tissue samples used as controls. RT-PCR and qPCR analysis confirmed these results. Similar results were obtained analysing FRA-1 expression in fine needle aspiration biopsy (FNAB) samples.

**Conclusion:**

The data shown here suggest that FRA-1 expression, including its intracellular localization, may be considered a useful marker for hyperplastic and neoplastic proliferative breast disorders.

## Background

The transcription factor complex AP-1 plays a central role in regulating gene transcription in several biological processes [1]. It is constituted of a great variety of dimers composed of members of the Fos, Jun, and ATF families of proteins. While the Fos proteins (c-Fos, FosB, Fra-1, Fra-2) can only heterodimerise with members of the Jun family, the Jun proteins (c-Jun, JunB, JunD) can both homodimerise and heterodimerise with Fos and ATF families members to form transcriptionally active complexes [2,3]. Several data show that modifications in AP-1 activity are correlated with cell transformation. In fact, cellular transformation and malignancy induced by *ras *genes require *c-jun*, and *fra-2/-c-jun *heterodimers that play a crucial role in chicken fibroblast transformation [4,5]. In particular, the induction of *fra-1 *seems to have a pivotal role in the process of cell transformation and in the carcinogenesis. Indeed, a) *fra-1 *exhibits oncogenic potential: its over-expression in rat fibroblasts stimulates anchorage-independent growth in absence of clear morphological transformation [6]; b) the inhibition of FRA-1 protein synthesis by stable transfection with a *fra-1 *antisense construct significantly reduces the malignant phenotype of the transformed thyroid cells [7]; c) FRA-1 induces morphological transformation and increases *in vitro *invasiveness and motility of epithelioid adenocarcinoma cells [8]; d) transfection with a dominant-negative *fra-1 *construct reverts the transformed phenotype of mesothelioma cells, inhibiting their anchorage-independent growth in soft agar [9]; e) ectopic co-expression of *c-jun *and *fra-1 *in NIH3T3 fibroblasts leads to a transformed phenotype [10]. High Fra-1 expression is associated with a more malignant cell phenotype, and suggesting that Fra-1 could have a pivotal role in breast cancer progression [11].

Consistently with this critical role of FRA-1 in cell transformation, an increased FRA-1 expression in neoplastic tissues has been frequently reported. In fact, a significant increase of FRA-1 protein levels was detected in all oncogene transformed rat thyroid cell lines, and also in human thyroid carcinoma cell lines and tissues [12]. Differential gene expression in non-malignant and malignant nasopharyngeal epithelial (NPE) cells, using a cDNA array hybridization method, has evidenced *fra-1 *expression only in the malignant cells [13]. Equally, the transcriptional profile of rat pancreatic carcinoma cell lines BSp73-AS (non-metastatic) and BSp73-ASML (highly metastatic) has revealed FRA-1 expression only in the metastatic cells, supporting a main role of *fra-1 *in the invasion process [14]. This seems to be confirmed by the silencing of *fra-1 *gene, showing that the induction of CD44 and c-met is causally linked to *fra-1 *expression, then connecting *fra-1 *with genes governing cell motility and invasion in mesothelioma [15].

In breast carcinomas, the analysis of the AP-1 family members (c-jun, junB, junD and c-fos, fosB, fra1 and fra2) revealed association of *fosB *expression with a well-differentiated receptor-positive tumor phenotype, whereas the expression of *fra-1 *showed significant negative correlation with *fosB *expression, as well as with estrogen-receptor status and differentiation [16]. Moreover, cDNA hybridization arrays to analyze the gene expression profiles (GEPs) of nine weakly invasive and four highly invasive breast carcinoma cell lines revealed *fra-1 *expression only in the highly invasive cell lines [17]. Therefore, also these results seem to support a critical role of *fra-1 *in breast carcinomas, likely in the progression step. On this basis, it was also conceived a vaccination versus the FRA-1 protein to prevent breast carcinomas. Indeed, the growth of primary subcutaneous tumor and dissemination of pulmonary metastases, induced by the inoculation of D2F2 murine breast carcinoma cell line, were markedly suppressed by a vaccine carried by attenuated Salmonella typhimurium and encoding murine FRA-1 [18].

Therefore, the aim of our study was to investigate whether evaluation of FRA-1 protein expression in breast neoplasias using different technical approaches might be a useful tool in the diagnosis and prognosis of human breast carcinomas. In the present study, we show that no *fra-1 *expression is detectable in normal breast tissue. Conversely, FRA-1 protein was abundant in all of the carcinoma samples with an intense staining in the nucleus. Positive staining was also found in fibroadenomas and hyperplasias without atypia, but in these cases the staining was both in the nucleus and cytoplasm, and the number of positive cells was lower than carcinomas. Conversely, atypical hyperplasias and *in situ *ductal carcinomas showed positivity restricted mainly to the nuclei. Analogous results were obtained analysing FRA-1 expression in fine needle aspiration biopsy (FNAB) samples.

## Methods

### Tissue samples

Tissue sections for immunohistochemistry [106 breast tissue specimens, constituted by 6 normal breast tissues corresponding to a macroscopically healthy region distinct from neoplastic lesions, 15 hyperplastic lesions (6 with atypical lesions), 15 fibroadenomas, 10 *in situ *ductal carcinomas, 40 ductal carcinomas and 20 lobular carcinomas] were obtained from routinely processed paraffin-embedded samples of breast neoplasias resected from the National Institute of Cancer G. Pascale foundation in Naples, Italy. Institutional ethical approval and informed consent will given by the patients undergoing surgery for breast cancer. The criteria for inclusion in the study were that the routinely processed paraffin blocks were suitable for immunohistochemistry, and adequate clinical information. The clinical and histological characteristics of the 60 carcinomas were: 51 patients (85%) presented with stage I and 9 patients (15%) with stage II tumours. 70% of tumors were hormonal receptor-positive and 30% were hormonal receptor-negative. Elston-Ellis grade determined at diagnosis was I in 12 patients (20%), II in 38 (63%) and III in10 patients (17%).

### Immunohistochemistry

5–6-μm-thick paraffin sections were deparaffinized and analysed as previously described [19]. Slides were incubated with the primary antibodies diluted 1:100 in PBS and then with biotinylated goat antirabbit IgG for 20 min (Vectostain ABC kits; Vector Laboratories), followed from the incubation with premixed reagent ABC (Vector) for 20 min. The immunostaining was performed by incubating slides in diaminobenzidine (Dako) solution containing 0.06 mM diaminobenzidine and 2 mM hydrogen peroxide in 0.05% PBS (pH 7.6) for 5 min. After chromogen development, slides were washed, dehydrated with alcohol and xylene, and mounted with coverslips using a permanent mounting medium (Permount). Micrographs were taken on Kodak Ektachrome film with a photo Zeiss system.

### Immunohistochemycal analysis of breast Fine Needle Aspiration Biopsies (FNAB)

The FNABs were performed at the "Istituto Nazionale dei Tumori di Napoli" in Italy, according to established procedures using a 25-gauge needle attached to a 20 ml syringe in a plastic syringe holder. Alcohol-fixed and air-dried smears were immediately immunostained using the same antibody as described above. Samples were obtained from 10 patients with breast neoplasias who subsequently underwent surgery because examination of the FNAB yielded cytologic diagnoses suspicious for cancer. Normal breast cells obtained after FNAB of the normal adjacent tissues of the patients were used as controls. The antibodies were made in our laboratory [20]. They are specific for FRA-1 and non-cross-reactive with the other members of the Fos family. The immunostained samples were blindly read by three independent individuals (G.C., M.D.B. and G.B.).

### Western blot

Immunoblottings were performed according to standard procedure, and previously described [12]. Anti-FRA-1 antibodies were previously described [20], and anti-tubulin was from Santa Cruz Biotechnology. Protein extract from a positive cell line (MDA-MB231) was loaded in one well as control for comparable exposition of chemiluminescent membranes.

### RT-PCR analysis

RNA was extracted from paraffin-embedded blocks on 40 cases analyzed in parallel for FRA-1 expression by immunohistochemistry, as previously described [18]. One μg of total RNA, digested with Dnase, was reverse transcribed using random exanucleotides as primers (2.5 μM) and 50 units of MuLV Reverse transcriptase, and subsequent PCR amplification was performed as mentioned here after. Two hundred ng of cDNA were amplified in a 25 μl reaction mixture containing 1.25 unit Taq DNA in polymerase buffer, 0.2 mM deoxynucleotide triphosphate, 1.5 mM MgCl_2_, 1.30 μM of each primer (Applied Biosystem-Roche). The PCR amplification was performed for 40 cycles (94°C for 30 s, 54°C for 30 s, and 72°C for 30 s). The specific primers for FRA-1 were: forward 5'-cgaaggccttgtgaacagat-3', and reverse 5'-ctgcagcccagatttctcat-3'; corresponding to the nucleotides 315–335 and 459–478, respectively. Amplification of contaminating genomic DNA was excluded by control experiments in absence of reverse transcriptase. Glyceraldehyde-3-phosphate-dehydrogenase (GAPDH) expression was used as internal control [12]. A PhosphorImager screen was briefly exposed to the membranes, and the screen was then scanned on a Molecular Dynamics PhosphorImager. The images recorded by the PhosphorImager were analyzed by volume integration with the ImageQuant software.

### Quantitative PCR

#### Selection of primers and probes

To design a qPCR assay, we used a Human ProbeLibray™ system (Exiqon, Denmark) [21,22]. The free ProbeFinder assay design software is an integrated part of the package. All fluorigenic probes were dual-labeled with FAM at 5' end and with a black quencer at the 3' end. We chose the best probe and primers pair to amplify a fragment for real-time PCR of *fra-1 *mRNA, entering its accession number (X16707) on the assay design page of the ProbeFinder software. Based on these data, the software provided us various solutions. We chose an amplicon of 119 nucleotides scattered among first and second exon. The number of probe was "human 27" (according to the numbering of Exiquon's Human ProbeLibry kit), and the primer sequences were: FRA-1 forward 5'-gggcatgttccgagacttc-3'; FRA-1 reverse 5'-gcaccaggtggaacttctg-3'. The same procedure was used to choose both probe and primers for housekeeping gene glucose 6-phosphate dehydrogenase *g6pd*, accession number X03674. The ProbeFinder provided us various solutions for *g6pd*, as well as *fra-1 *transcript. We opted for an amplicon of 106 nucleotides scattered among third and fourth exon. The number of probe was "human 05", and the primer sequences were: *g6pd *forward 5'-acagagtgagcccttcttcaa-3'; *g6pd *reverse 5'-ggaggctgcatcatcgtact-3'.

#### cDNA preparation

1 μg of total RNA of each sample was reverse-transcribed with the RNA PCR Core kit (Applied Biosystems, Roche) using random hexamer primers according to the manufacturer's instructions.

## Results

### Immunohistochemical analysis of FRA-1 expression in breast neoplastic samples

**Figure 1:**
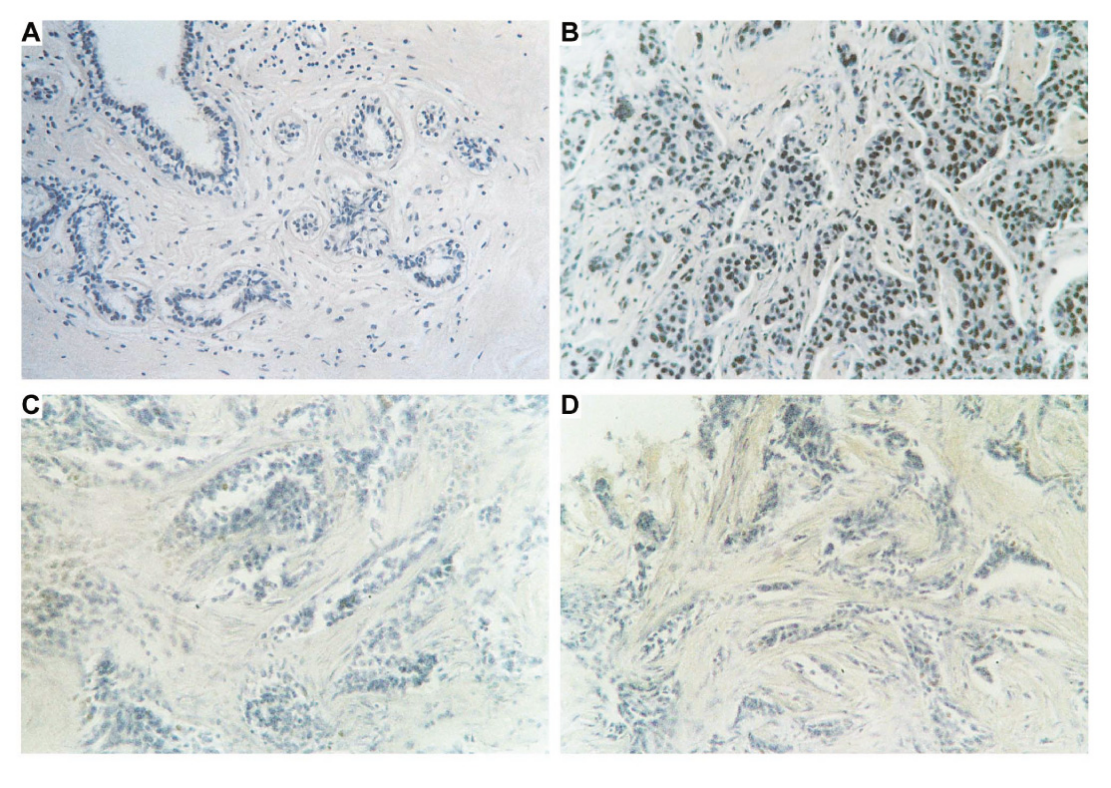
**Immunohistochemical analysis of FRA-1 protein in breast tissues**. Immunostaining of a normal breast tissue (100 ×) **(A) **and of a ductal carcinoma (200 ×) **(B)**. The same ductal carcinoma was immunostained in the absence of the primary antibody (200 ×) **(C)**, or with the FRA-1 antibodies pre-incubated with the peptide against which the antibodies were raised (200 ×) **(D).**

**Figure 2:**
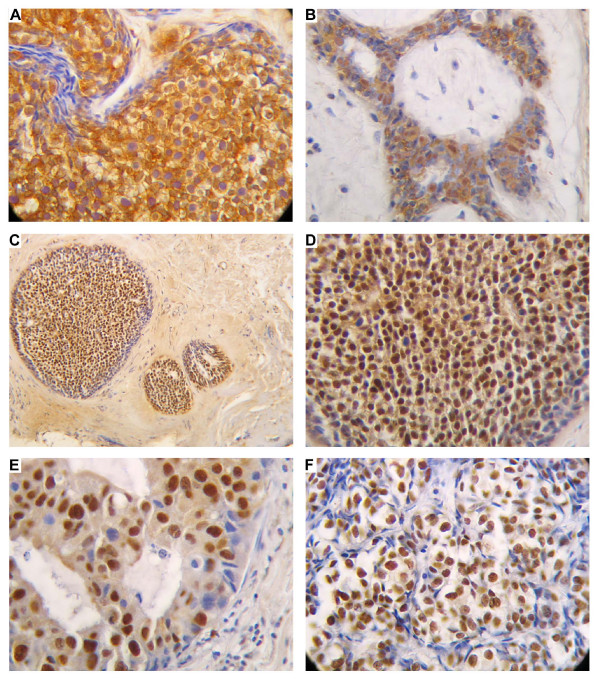
**Immunohistochemical analysis of FRA-1 in benign and malignant breast tissues**. Paraffin sections from hyperlastic and neoplasic breast tissues were analyzed by immunohistochemistry using antibodies raised against a specific FRA-1 peptide.**A) **Nuclear and cytoplasmic staining in a typical breast hyperplasia (200 ×). **B) **Immunostaining of a breast fibroadenoma (200 ×) showing nuclear and cytoplasmic staining. **C) **and **D) **Immunostaining of an atypical hyperplasia at two different magnification (100 ×) and (200 ×) respectively: the staining is present in the majority of the nuclei and is weakly present in the cytoplasm. **E) **Immunostaining of a ductal *in situ *breast carcinoma (400 ×): the immunostaining is restricted to the nucleus and weakly present in the cytoplasm, but it is not present in all the cells. **F) **Immunostaining of a lobular carcinoma (400 ×): the immunostaining is restricted to the nucleus, and is present in all malignant cells.

The immunohistochemical technique was chosen for FRA-1 protein analysis because it allows a rapid and sensitive screening of breast pathological tissues and is amenable to regular use as a routine diagnostic test. We used antibodies raised against a FRA-1 specific peptide generated in our laboratory [20], to analyse 106 breast tissues, including 6 normal tissues, 15 dysplasias (6 with atypical lesions), 15 fibroadenomas, 10 *in situ *ductal carcinomas, 40 ductal carcinomas and 20 lobular carcinomas. The results of the immunohistochemical study of breast specimens are summarized in Table 1. Most of the breast carcinoma samples (92%) showed a strong nuclear immunoreactivity in the majority of the cells. No cytoplasmic staining was observed. Similar results were obtained when *in situ *ductal carcinomas were analyzed, even though the immunoreactivity was restricted to a reduced number of cells in comparison to the carcinoma samples and a weak cytoplasmic staining is present. As far as breast hyperplasias are concerned, we observed no different FRA-1 protein levels between atypical and typical breast hyperplasias, but the FRA-1 immunolocalization was essentially present in the nuclei in the atypical lesions, whereas the typical lesions showed a mainly cytoplasmic staining. Conversely, no staining at all was observed in six normal breast tissues. Positive staining was also found in benign fibroadenomas, but, in this case, the staining was present both in the nucleus and cytoplasm, and these samples showed a weaker staining in comparison to the breast carcinomas. In the Figures 1 and 2, we show some representative immunohistochemical analyses. No positive signal is detected in normal breast tissue (Figure 1A), whereas an intense nuclear staining is clearly observed in a ductal carcinoma (Figure 1B). The specificity of the antibodies versus the FRA-1 protein was also evaluated. In fact, for each case, sections were stained without the primary antibody. In all cases these controls were negative (Figure 1C). Equally, no staining was also observed when the neoplastic tissues were analyzed after that the FRA-1 specific antibodies have been pre-incubated with the FRA-1 control peptide (Figure 1D). Figures 2A and 2B show the immunostaining of a positive typical hyperplasia and a positive fibroadenoma, respectively: a minority of the cells show nuclear immunoreactivity that appeared associated with a constant, cytoplasmic staining. In the case of the *in situ *ductal breast carcinomas, the staining was always restricted to the nucleus, but it was not detected in all the cells (Figure 2E). Figures 2C and 2D show the immunostaining of an atypical hyperplasia at two different magnifications: the FRA-1 immunoreactivity is present in the majority of the nuclei and a cytoplasmic staining is present. This pattern of FRA-1 protein expression and localization is very similar to that observed in the breast *in situ *ductal carcinomas. Figure 2F show the immunostaining of a lobular breast carcinoma with an intense nuclear staining in all the malignant cells.

### Fra-1 overexpression in breast tumour samples

**Figure 3:**
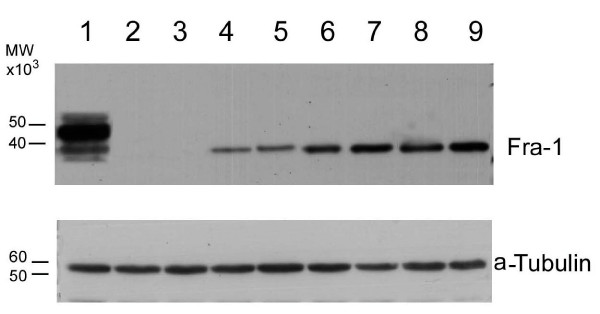
**Analysis of FRA-1 protein expression by western blot**. The imunoblotting analysis was performed on extracted proteins (20 μg/lane) in MDA-MB231 cells and in a limited number of breast samples, from normal, dysplastic and neoplastic breast tissues. Sources of the proteins: lane 1: MDA-MB231 cells; lanes 2–3: normal breast tissues; lanes 4–5: breast dysplastic lesions; lanes 6–9: breast cancer tissues.

In a limited number of samples ((2 normal breast tissues, 2 hyperplasias, and 4 ductal carcinomas), FRA-1 protein expression was also evaluated by Western blot analysis, that essentially confirmed the immunohistochemical data. In fact, as it is shown in the Figure 3, a FRA-1 specific single band with apparent molecular weight of 38,000 kDA was present in the carcinoma samples (lanes 6, 7, 8, 9) but not in the normal breast tissues (lanes 2, 3). Breast hyperplastic lesions showed FRA-1 protein expression but it was lower than that found in the carcinoma samples (lanes 4, 5). Specific FRA-1 protein bands were identified by apparent molecular weight relative to standard proteins (prestained protein ladder, Invitrogen). The presence of a single band in all samples would suggest that Fra-1 phosphorylation status does not change.

### Semiquantitative and quantitative RT-PCR analysis

**Figure 4:**
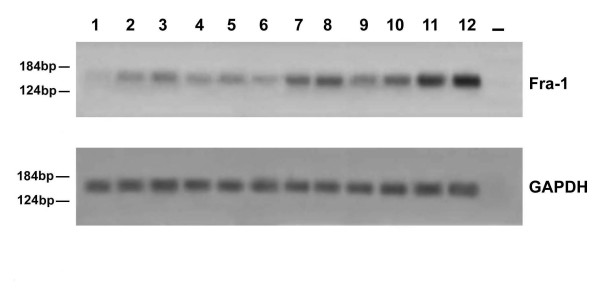
**RT-PCR analysis of the *fra-1 *gene expression in paraffin-embedded tissues**. The mRNA was extracted from paraffin-embedded tissues and amplified by RT-PCR using *fra-1 *specific primers as specified under "Material and Methods". The cDNAs were co-amplified with *gapdh *gene as an internal control. Sources of RNA: lane 1, normal breast tissue; lanes 2–4, breast dysplastic lesions; lanes 5–7, breast fibroadenomas; lanes 8–12, breast carcinomas; lane 13: water control. Molecular markers are indicated.

To quantitate the levels of the *fra-1 *gene expression and validate the immunohistochemical data, we also analyzed 26 samples (2 normal breast tissues, 8 hyperplasias, 6 fibroadenomas, and 10 ductal carcinomas) for the specific *fra-1 *mRNA levels by a semiquantitative RT-PCR assay. The results are consistent with the immunohistochemical findings. A representative RT-PCR assay is shown in Figure 4. The normal breast tissue samples were negative for *fra-1 *gene expression (lane 1). Three hyperplasias (lanes 2, 3 and 4) and three fibroadenomas (lanes 5, 6 and 7) were positive for *fra-1 *expression, at levels comparable among them. All of the malignant tumours showed a significant *fra-1 *expression (from lane 8 to 12). *Gapdh *amplification was used as internal control.

**Figure 5:**
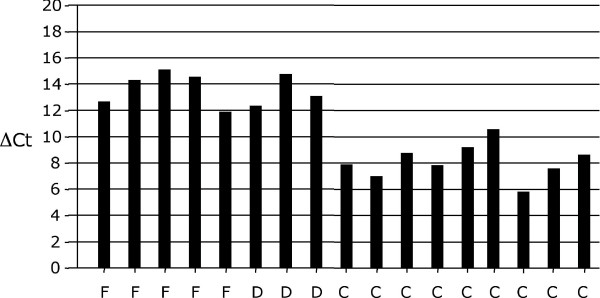
ΔCt, difference between Ct value of *fra*-1 mRNA and Ct value of *g6pd* mRNA (ΔCt =Ct*fra*-1-Ct*g6pd*). F, fibroadenoma; D, displasia; C, carcinoma.

To better evaluate the differences in *fra-1 *gene expression in the different breast samples, we performed a quantitative RT-PCR assay. To evaluate gene expression level, we used the ΔCt. For each sample, *fra*-1 Ct value was subtracted from *g6pd *Ct value (Ct_*fra*-1_-Ct_*g6pd*_), used to normalize the amount of cDNA. In Figure 5, we compared the ΔCt values of fibroadenoma, hyperplasia, carcinoma and normal breast samples. In breast carcinomas *fra*-1 Δct values (5.79–10.58) was lower because *fra*-1 mRNA could be detected at a number of cycles a little higher than that occurring for *g6pd *amplification. In fibroadenomas and hyperplasias the ΔCt values (11.91–15.10) were between the ΔCt of normal breast and ΔCt of carcinoma samples. The result of t-test (p-value = 0,00032) between ΔCt values of carcinomas group and ΔCt values of fibroadenomas and hyperplasias group indicated that there is a significant difference of *fra*-1 mRNA level between these tumor histotypes.

### Analysis of *fra-1 *gene expression in breast fine needle aspiration biopsy

**Figure 6:**
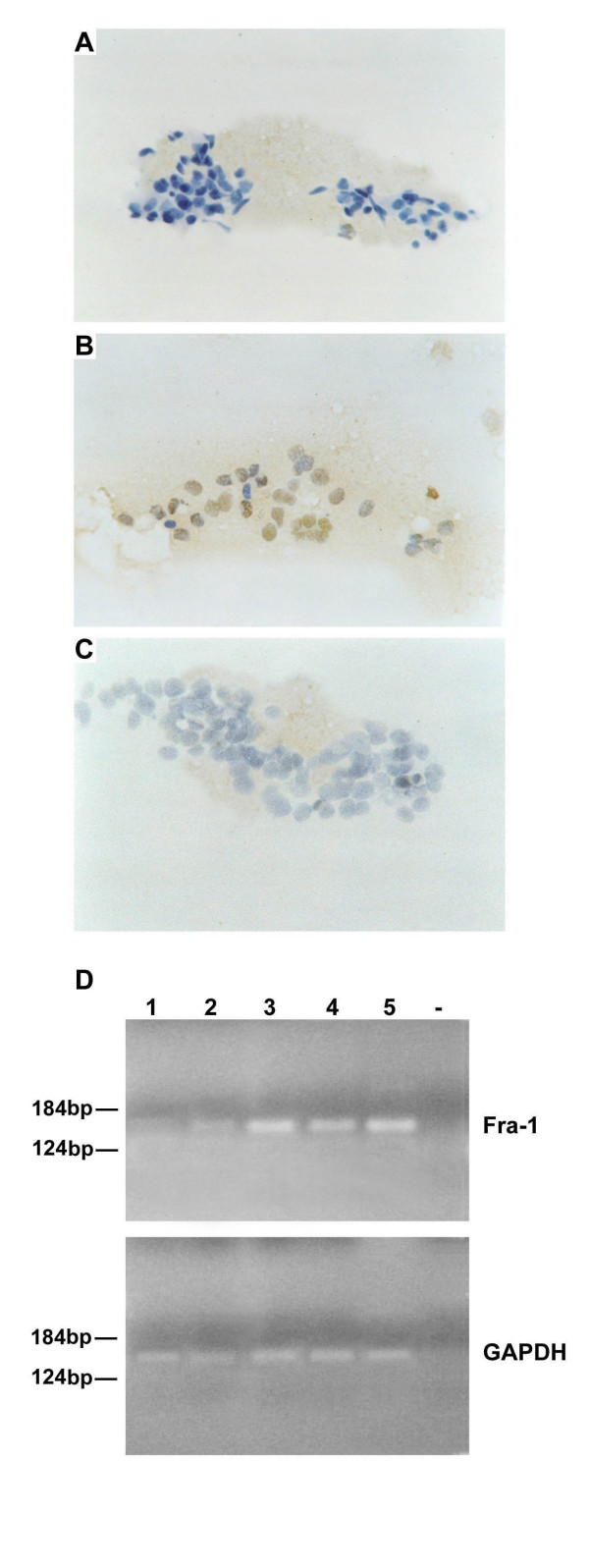
**Immunohistochemical and RT-PCR analysis of the *fra-1 *gene expression in fine needle aspiration biopsy samples (FNAB)**. The cytological specimens investigated by immunohistochemistry were also analyzed by RT-PCR. The mRNAs were extracted from FNABs of breast tissues and amplified by RT-PCR using *fra-1 *specific primers as specified under "Methods". The cDNAs were co-amplified with *gapdh *gene as an internal control. A) Immunostaining in a FNAB deriving from a normal breast tissue (100 ×), and from a carcinoma samples (100 ×) (B). C) A carcinoma FNAB was incubated without the primary antibody (100 ×) as control of the specificity of the antibodies. D) *Fra-1 *expression was analyzed by RT-PCR. The sources of RNAs were: lane 1: normal breast; lane 2, breast dysplasia; lanes 3, 4 and 5: breast carcinomas; lane -, water control. Molecular markers are indicated.

Fine needle aspiration biopsy (FNAB) has a critical role in the preoperative diagnostic analysis of breast neoplasias. To evaluate the applicability of *fra-1 *gene expression analysis to FNABs samples, we studied 10 cases of breast carcinomas and 4 normal breast samples obtained after FNAB of the normal adjacent tissues. In one patient (lane 3), we also obtained hyperplastic cells after FNAB from a hyperplastic region adjacent to the carcinoma. The cytological specimens were analyzed for *fra-1 *expression by immunohistochemistry and RT-PCR. The results are shown in Table 2 and Figure 6. Immunohistochemistry showed nuclear staining in the carcinoma samples (Panel B), but not in the normal tissue (Panel A). No staining was observed when the FNAB deriving from carcinoma was incubated in the absence of the primary antibody (Panel C). Equally, RT-PCR analysis (Panel D) revealed *fra-1 *gene expression in the carcinoma samples (lanes 3, 4, 5) but not in the normal breast tissue (lane 1) deriving from the same patient shown in lane 3. *fra-1 *expression was at a significant lower level in the hyperplastic region (lane 2) of the same patient shown in lane 3 in comparison to the carcinoma samples.

## Discussion

Breast cancer is the most common cancer and the second leading cause of cancer mortality in women, since epidemiologic data suggest that one in every eight women will suffer from breast carcinoma [23]. Breast neoplastic diseases range from benign fibroadenoma to very aggressive undifferentiated carcinoma. Growth factors and their receptors, intracellular molecules, regulators of cell cycling, and proteases, have all been shown to be altered in sporadic breast cancer [24], and c-Erb-B2 and HMGA1 overexpression and loss of estrogen receptors correlate with a poor prognosis [25].

Our aim was to verify whether FRA-1 protein detection might be useful in the diagnosis and prognosis of human breast neoplasias. The rationale for this study derives from several studies demonstrating a critical role of AP-1 complex, and, in particular, of one of its members FRA-1 in cell transformation, since FRA-1 promotes cell motility by inactivating beta-1 integrin and keeping RhoA activity low [26]. Moreover, recent data demonstrate that *fra-1 *expression is controlled by different thresholds of ERK activity, that is frequently increased in cancer. In fact, a basal ERK activity is required to induce transcription of the *fra-1 *gene, but additional higher levels of activity stabilize *fra-1 *against proteasome-dependent degradation [27]. In our study, we found that *fra-1 *expression, evaluated by immunohistochemistry, western blot and semiquantitative and quantitative RT-PCR, started to become detectable in fibroadenomas and hyperplastic stage and strongly detectable in carcinoma samples. Conversely, breast normal tissues did not show any detectable expression of *fra-1*. The gene expression level evaluated by qPCR indicated more than 40-fold difference between carcinomas group and fibroadenomas and hyperplastic group. In fact, the expression was higher and concerning the vast majority of the cells, and exclusively nuclear in the cancer tissues, whereas breast fibroadenomas and typical hyperplasias showed a weaker expression, not present in the majority of the cells, with a constant cytoplasmic staining. Concerning breast atypical hyperplasias, we did not observe different levels of FRA-1 protein expression compared the typical ones, but the FRA-1 immunolocalization was essentially present in the nuclei in the atypical lesions and a weakly cytoplasmic staining was present. A similar behavior was observed in "*in situ*" ductal carcinomas. In fact, the staining was restricted to the nucleus, but the number of positive cells was lower compared to the carcinomas samples. It is likely that this different behaviour of FRA-1 in these samples might be dependent on different genetic alterations. However, in contrast with previously published data showing a negative correlation between *fra-1 *expression and hormone receptor status in mammary carcinoma cell lines, no significant differences were observed in the FRA-1 expression among different morphological grading of the breast carcinoma samples and other prognostic indicators of breast carcinomas (ER and PgR receptors, Ki67 and ERB-B2), Therefore, its detection does not seem to have any prognostic value. It is more likely that the different immunolocalization and gene expression level of FRA-1 might represent a useful tool for differentiating breast carcinomas for their aggressiveness.

## Conclusion

It is noteworthy that the analysis of FNAB samples by immunohistochemistry and RT-PCR confirms the results obtained by the same techniques on the surgically removed tissues, suggesting that a preoperative evaluation of FRA-1 expression might be a useful tool in the diagnosis of breast dysplastic and/or neoplastic lesions. Moreover, the restriction of *fra-1 *expression to breast neoplastic tissues together with the previous results, indicating a critical role of *fra-1 *in the process of carcinogenesis, makes the FRA-1 protein as an excellent target for cancer therapy.

## Abbreviations

FRA-1 = Fos-related antigen 1; AP-1 = Activating protein-1; FNAB = fine needle aspiration biopsy; NPE = nasopharyngeal epithelial cells; GEPs = gene expression profiles; qPCR = quantitative polymerase chain reaction; GAPDH = Glyceraldehyde-3-phosphate-dehydrogenase; G6PD = glucose 6-phosphate dehydrogenase gene; HMGA = High mobility group A; ER = estrogen receptor; PgR = progesteron receptor.

## Competing interests

The author(s) declare that they have no competing interests.

## Authors' contributions

GC participated in the design of the study, analyzed the immunohistochemical data and helped to draft the manuscript. AF carried out the q-PCR and analyzed data. GB and MBD performed the pathology analysis for each tumor and analyzed the immunohistochemical data. GDA was the surgeon who provided the breast tumors as well as the fine needle aspiration biopsies. RP and EV isolated RNAs and performed RT- PCR analysis. MM performed the immunohistochemical analysis. GMP and LA isolated proteins and performed Western Blot analysis. AF conceived the study, participated in its design and drafted the manuscript.

## Pre-publication history

The pre-publication history for this paper can be accessed here:



## Figures and Tables

**Table 1:** FRA-1 protein expression and localization in normal and neoplastic breast tissues by immunohistochemistry.

	FRA-1 staining score* N(%)					FRA-1 localization**		
	
**Histological type**	**0**	**1+**	**2+**	**3+**	**4+**	**N**	**C**	**N/C**
Normal breast tissues (N = 6)	6 (100)	0 (0)	0 (0)	0 (0)	0 (0)	0	0	0
Fibroadenomas (N = 15)	0 (0)	2(13)	13(87)	0(0)	0(0)	2	11	2
Typical ductal hyperplasias (N = 9)	0 (0)	3 (33)	5 (56)	1 (11)	0 (0)	1	2	6
Atypical ductal hyperplasias (N = 6)	0 (0)	1 (17)	3 (50)	2 (33)	0 (0)	4	1	1
*In situ *ductal carcinomas (N = 10)	0 (0)	1 (10)	7 (70)	2 (20)	0 (0)	6	1	2
Ductal + Lobular carcinomas (N = 60)	0 (0)	0 (0)	(0)	5 (8)	55(92)	52(0)	0 (0)	8

*FRA-1 staining score was: 0, no. positive cells; +, positive cells; 1+, < 10% of positive cells; 2+, 11–50% of positive cells; 3+, 51–75% of positive cells; 4+, 76–100% positive cells. N, number of cases.

**N=Nuclear, C = Cytoplasmic, N/C = nuclear and cytoplasmic staining

**Table 2:** Analysis of FRA-1 protein expression in ductal breast FNABs

Cytological Diagnosis	N° of positive cases/number of cases analysed by immunohistochemistry	N° of positive cases/number of cases analysed by RT-PCR	Histological Diagnosis
Normal tissues	0/4	0/4	Normal breast
Ductal breast carcinomas	8/8	8/8	Ductal breast carcinomas
Lobular breast carcinomas	2/2	2/2	Lobular breast carcinomas
